# CD93 and dystroglycan cooperation in human endothelial cell adhesion and migration

**DOI:** 10.18632/oncotarget.7136

**Published:** 2016-02-02

**Authors:** Federico Galvagni, Federica Nardi, Marco Maida, Giulia Bernardini, Silvia Vannuccini, Felice Petraglia, Annalisa Santucci, Maurizio Orlandini

**Affiliations:** ^1^ Department of Biotechnology, Chemistry and Pharmacy, University of Siena, 2-53100 Siena, Italy; ^2^ Department of Molecular and Developmental Medicine, Obstetrics and Gynecology, University of Siena, 53100 Siena, Italy

**Keywords:** angiogenesis, signal transduction, C1qRp, Src, Cbl

## Abstract

CD93 is a transmembrane glycoprotein predominantly expressed in endothelial cells. Although CD93 displays proangiogenic activity, its molecular function in angiogenesis still needs to be clarified. To get molecular insight into the biological role of CD93 in the endothelium, we performed proteomic analyses to examine changes in the protein profile of endothelial cells after CD93 silencing. Among differentially expressed proteins, we identified dystroglycan, a laminin-binding protein involved in angiogenesis, whose expression is increased in vascular endothelial cells within malignant tumors. Using immunofluorescence, FRET, and proximity ligation analyses, we observed a close interaction between CD93 and β-dystroglycan. Moreover, silencing experiments showed that CD93 and dystroglycan promoted endothelial cell migration and organization into capillary-like structures. CD93 proved to be phosphorylated on tyrosine 628 and 644 following cell adhesion on laminin through dystroglycan. This phosphorylation was shown to be necessary for a proper endothelial migratory phenotype. Moreover, we showed that during cell spreading phosphorylated CD93 recruited the signaling protein Cbl, which in turn was phosphorylated on tyrosine 774. Altogether, our results identify a new signaling pathway which is activated by the cooperation between CD93 and dystroglycan and involved in the control of endothelial cell function.

## INTRODUCTION

Endothelial cell (EC) migration is a complex process fundamental to angiogenesis. During cell migration, the integration of several signals, largely provided by extracellular matrix (ECM), is essential to activate intracellular signaling pathways that affect cell shape, polarity, and cytoskeletal remodeling [1]. Several classes of cell surface proteins have been demonstrated to play important roles in the regulation of EC migration and blood vessel formation [2]. In addition to integrins, a family of adhesion receptors responsible for high affinity adhesion to ECM, other surface molecules can bind ECM components and induce signaling [3]. Therefore, the discovery of alternative adhesion mechanisms and their contribution to angiogenesis may shed light on the molecular machinery of blood vessel formation.

CD93, also known as the complement component C1q receptor (C1qRp), is a cell surface glycoprotein, which together with endosialin and thrombomodulin constitute a small family of transmembrane proteins with similar extracellular domains [4]. In our previous report [5], we demonstrated that CD93 activated angiogenesis by promoting adhesion of ECs. Along with our findings, a good amount of evidence suggests that CD93 plays a role in the endothelium. Indeed, its predominant site of expression is the vascular endothelium and, in the developing embryo, the mouse homologue of CD93 is expressed especially during the remodeling of blood vessels [6, 7]. Furthermore, the surface domains of CD93, which are susceptible to protein ectodomain cleavage, act as angiogenic growth factors [8].

Dystroglycan (DG) is an important ECM adhesion molecule first isolated from skeletal muscle as a component of the dystrophin glycoprotein complex [9, 10]. DG consists of two non-covalently associated subunits (α and β) encoded from a single gene and binds to several ECM ligands [11]. DG is widely expressed and, even though its role in the conservation of muscle integrity has been well established, it has a more general function in cell adhesion and signaling that is not yet fully understood [12]. Indeed, DG is expressed in ECs and involved in cell adhesion to ECM [13–15]. Moreover, it was observed that β-DG was needed for EC migration on laminin matrix and its expression was downregulated in quiescent and differentiated ECs. On the contrary, β-DG expression level was found to be high in ECs of malignant tumors, hyperplastic and inflamed tissues, where ECs were assembling new blood vessels [14, 16]. Although these observations strengthen the involvement of CD93 and DG in angiogenesis, their molecular mechanisms of action in the endothelium are largely unknown.

In the present study, we identify cooperative interactions between CD93 and DG and demonstrate that this relationship promotes EC migration, survival and tube formation. We show that phosphorylation of CD93 through DG engagement is instrumental in EC migration and differentiation, unveiling new signaling mechanisms that govern EC migration in the context of angiogenesis.

## RESULTS

### CD93 and β-DG exhibit a close association in ECs

**Figure 1:**
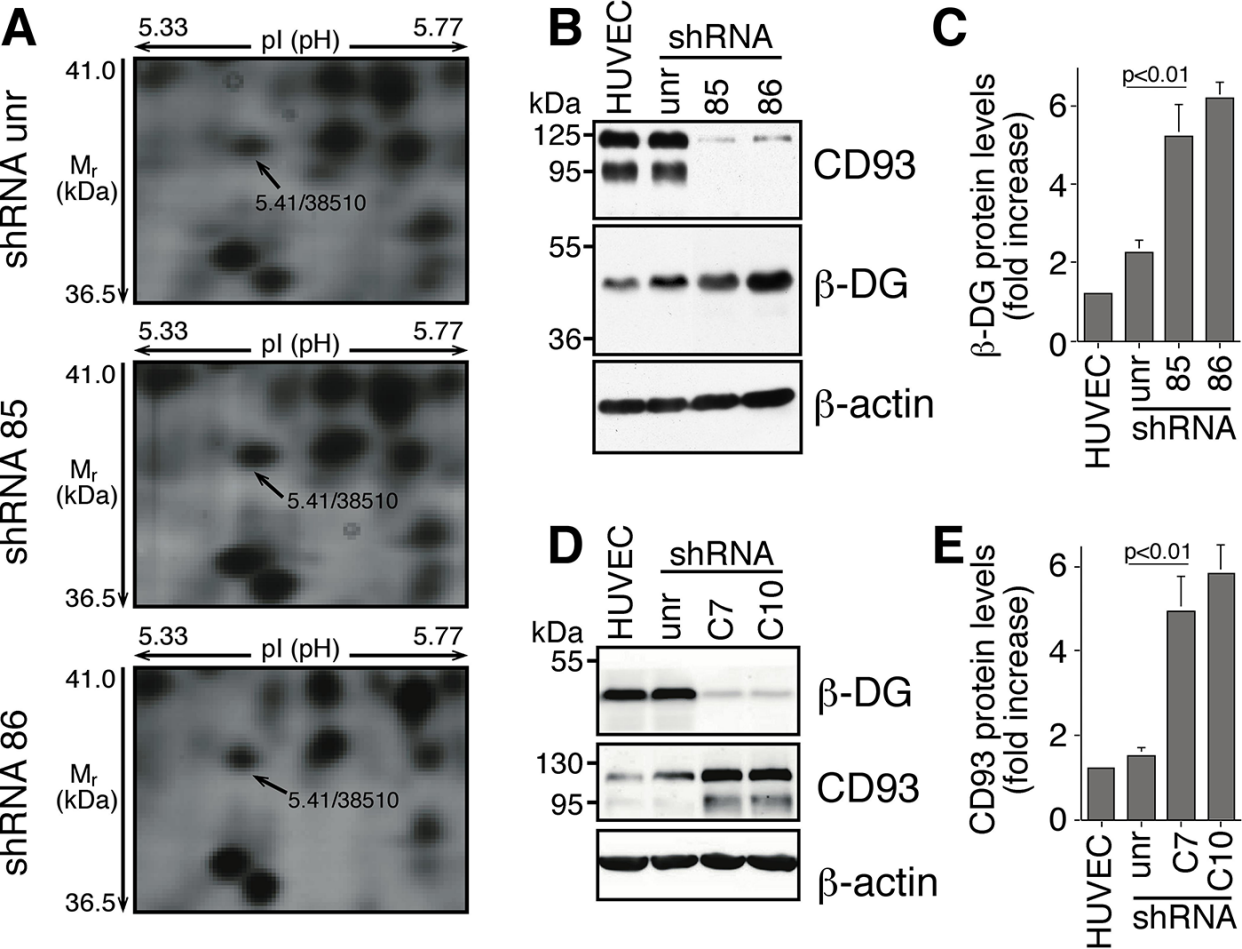
In ECs DG and CD93 silencing reveals adaptive changes in expression of both proteins (**A**) HUVEC were infected with lentiviral vectors expressing unrelated (unr) or CD93 shRNAs (clones 85 and 86). Total cell lysates were obtained from exponentially growing cells and subjected to comparative proteomic analysis. Expanded views from the 2-DE gels show the increased expression pattern of β-DG in CD93-silenced cells compared to cells expressing an unrelated shRNA. Arrows indicate the experimental coordinates (p*I* and M_*r*_) of the spot identified as β-DG by mass spectrometry. (**B**) HUVEC were infected as in A. Cell extracts were analyzed by Western blotting using anti-CD93 and anti-β-DG antibodies. Anti-β-actin antibodies were used to confirm equal protein loading. Not infected cells (HUVEC). (**C**) Quantitative analysis of β-DG protein levels from independent experiments performed as in B. Protein levels were quantified by densitometric scanning and the values, normalized to β-actin protein levels, were averaged and expressed as arbitrary units. (**D**) ECs were infected with lentiviral vectors expressing unrelated (unr) or DG shRNAs (clones C7 and C10). Cell extracts were analyzed by Western blotting as in B. (**E**) Quantitative analysis of CD93 protein levels performed as in D, normalized to β-actin protein levels, and expressed as arbitrary units. Representative images from a triplicate set are shown and data represent the means ± SD of three independent experiments.

To get molecular insight into the characterization of the biological role of CD93 in the endothelium, we performed proteomic analyses to examine changes in the protein profile of human umbilical vein ECs (HUVEC) after CD93 knockdown by lentiviral expression of small hairpin RNAs (Figure S1). By comparing 2-dimensional electrophoresis (2-DE) maps obtained from ECs following manipulation of CD93 expression and cells expressing an unrelated shRNA, we identified 52 differentially expressed proteins (data not shown). Among these proteins, the cell adhesion molecule β-DG appeared upregulated (Figure 1A). This upregulation was validated by Western blotting analysis, which showed that in ECs, β-DG levels increased following CD93 knockdown (Figure 1B and 1C). Since in the cellular machinery of adhesion, a specific cell adhesion molecule may influence the activity and/or expression of another type of molecule [17–19], we further investigated the effects of DG silencing on CD93 protein levels. β-DG protein levels were decreased in HUVEC by the use of two lentiviral constructs expressing two independent DG shRNAs (clones C7 and C10). HUVEC infected with lentiviruses expressing either DG shRNA showed reduced β-DG but increased CD93 protein levels as compared to non-infected cells or cells infected with a lentivirus expressing an unrelated shRNA (Figure 1D and 1E), suggesting the existence of a bidirectional connection between CD93 and β-DG in ECs.

**Figure 2:**
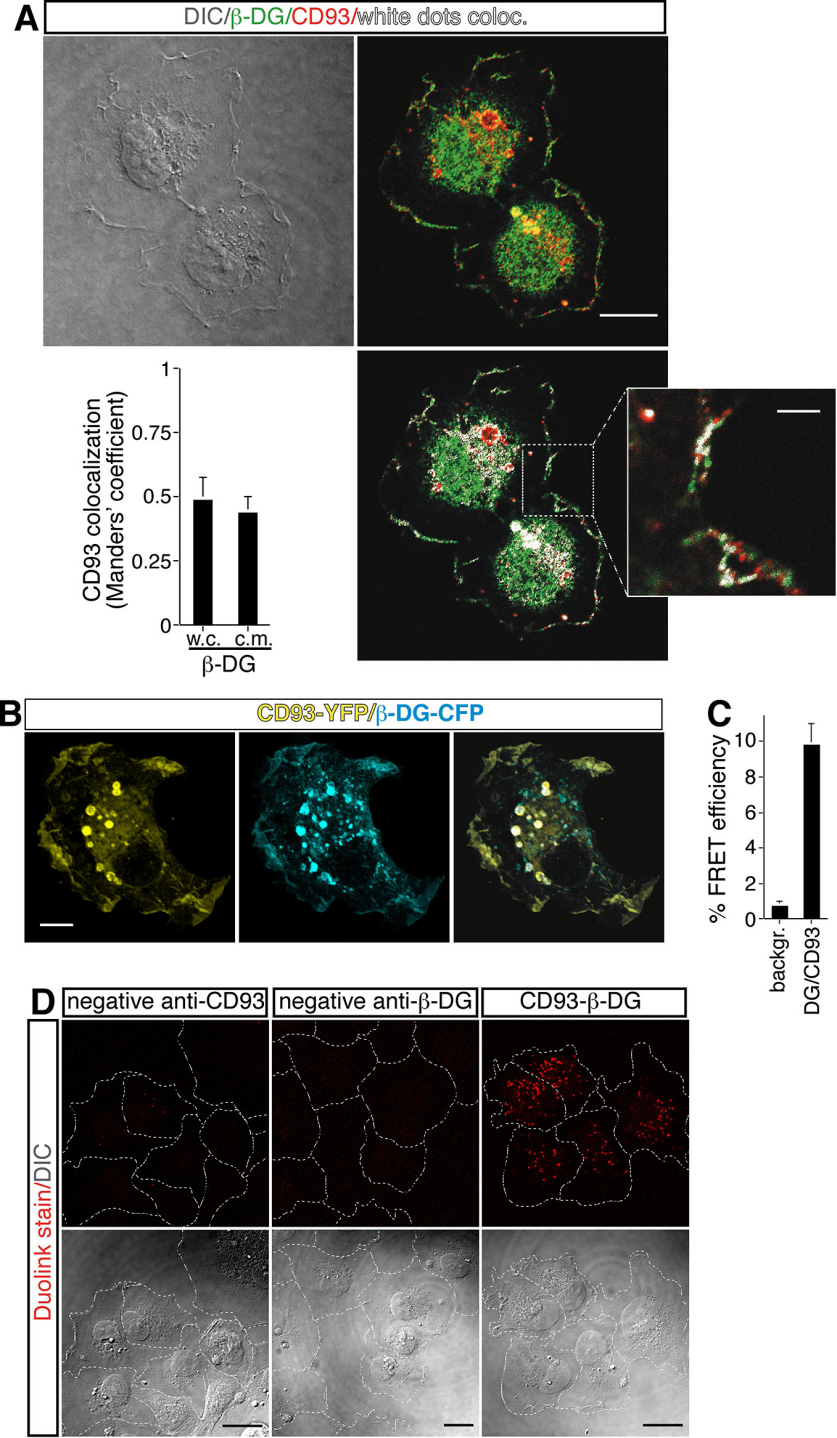
Direct association between CD93 and β-DG in ECs (**A**) HUVEC, plated onto laminin-coated glass coverslips, were fixed during the late phase of spreading and analyzed by immunofluorescence using anti-β-DG and anti-CD93 antibodies. Differential interference contrast (DIC), overlay of stained cells, and white dot colocalization images are shown. Plot shows quantification (using Manders' coefficient) of CD93 colocalization with β-DG at the cell margin (c.m.) and in whole cells (w.c.) (mean ± SD; cells = 21; *n* = 3). Scale bar, 12 μm. In the inset white dots show CD93 and β-DG colocalization at the cell margin. Scale bar of the inset is 3 μm. (**B**) CD93-YFP and β-DG-CFP were cotransfected into ECs. Fully spread cells on laminin-coated surfaces were fixed and subjected to immunofluorescence. Immunofluorescence shows CD93 and β-DG colocalization both at the plasma membrane and within intracellular vesicles. Scale bar, 8 μm. (**C**) Cells treated as in B were subjected to FRET analyses. The mean value of the FRET efficiency between acceptor (CD93-YFP) and donor (β-DG-CFP) was 9.11 ± 0.84%, after subtraction of the background. FRET data represent the means ± SD of three independent experiments, carried out on different days and with different cell preparations. (**D**) Representative confocal images of CD93/β-DG protein interaction detected *in situ* by Duolink stain. HUVEC exponentially growing on laminin-coated surfaces were fixed and treated at the same time with mouse anti-CD93 and rabbit anti-β-DG antibodies (CD93-β-DG). Close proximity of the primary antibodies was revealed by localized amplification. Protein-protein interactions were visualized as individual spots by red fluorescence. Background was assayed by removing one of the two primary antibodies from the reaction (anti-β-DG antibodies removed, neg. contr. CD93; anti-CD93 removed, neg. contr. β-DG). DIC images of stained cells are shown. The corresponding cell boundary is indicated by white dotted lines. Experiment was performed three times. Scale bars represent 18 μm.

To assess the hypothesis that CD93 and DG can interact together, double immunofluorescence staining and colocalization analysis of fully spread ECs showed that CD93 and β-DG shared a similar cellular labeling pattern and displayed significant colocalization at cell surface (Figure 2A). To verify whether CD93 and β-DG interacted directly we transiently transfected CD93-YFP and β-DG-CFP into HUVEC and performed fluorescence resonance energy transfer (FRET) analysis. Transfected cells were initially analyzed by immunofluorescence using anti-CD93 and anti-β-DG antibodies to confirm that the exogenously expressed proteins were indeed CD93 and β-DG (Figure S2 and [5]). Next, we investigated CD93-YFP and β-DG-CFP cellular localization. In ECs adhering to the substrate, both tagged proteins showed a high degree of colocalization at the cell membrane and within intracellular vesicles (Figure 2B). Afterward, in cells transfected with both expression plasmids, photobleaching of the acceptor fluorophor (CD93-YFP) increased the fluorescence intensity of the donor (β-DG-CFP) (Figure 2C), indicating that the two fluorophores were in close proximity. After photobleaching, additional ROIs, including more discrete cell regions both inside and outside the bleached area, were chosen for a more accurate evaluation of FRET efficiency and background (Figure S3).

To assess if the interaction was direct as suggested by the FRET analyses, we performed a proximity ligation assay, which allows *in situ* localization of protein-protein interactions at single-molecule resolution [20]. In exponentially growing ECs treated simultaneously with anti-CD93 and anti-β-DG primary antibodies, we observed the presence of fluorescent spots due to localized amplification of the probes bound in close proximity, whereas we did not observe any fluorescent signal when the primary antibodies were used alone (Figure 2D). Altogether, these results support the idea that in ECs CD93 and β-DG are in close association.

### CD93 or DG silencing impairs EC function

**Figure 3:**
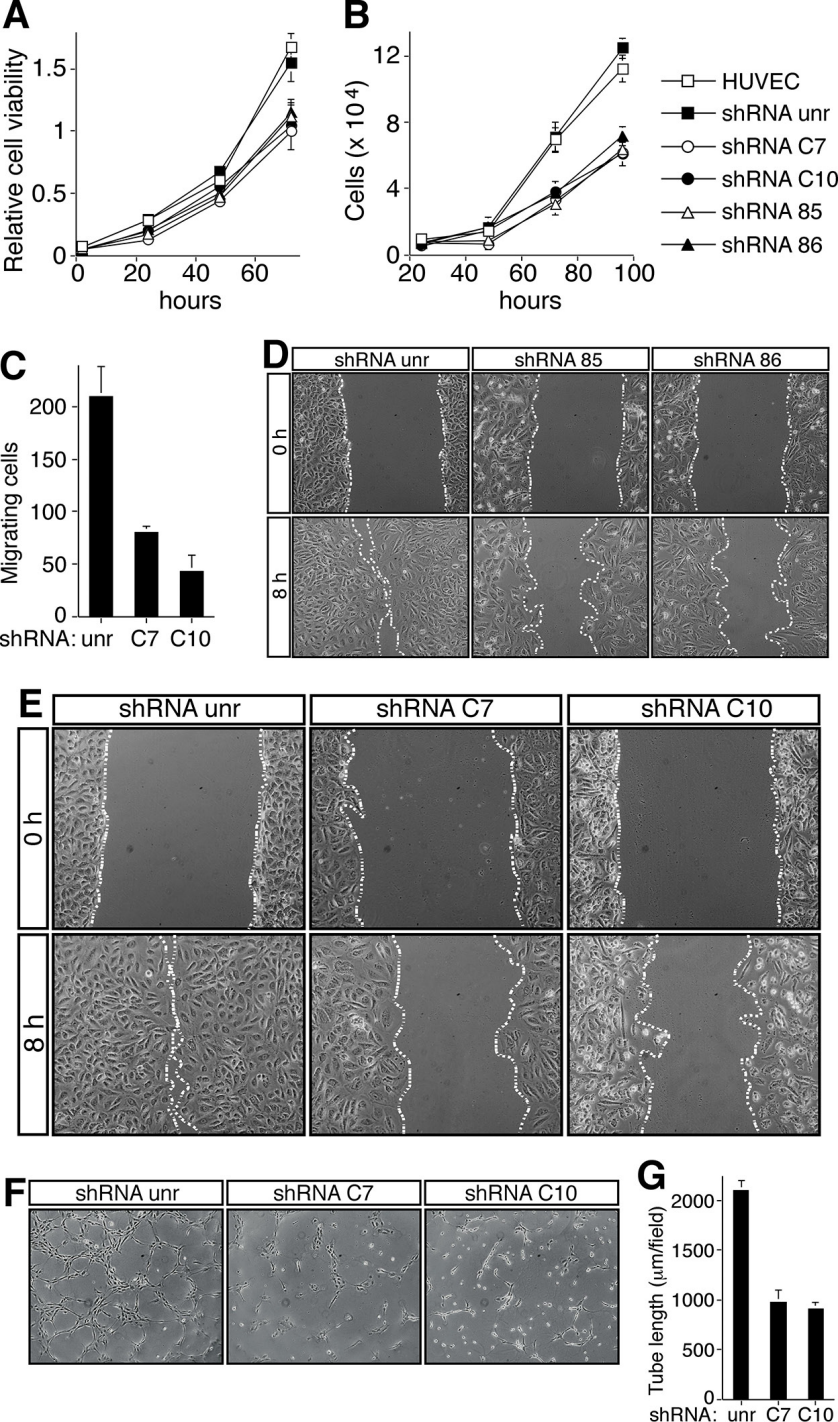
CD93 or DG knockdown impairs EC function HUVEC were infected with a lentiviral vector expressing unrelated (unr), or CD93 (clones 85 or 86), or DG (clones C7 or C10) shRNAs. Not infected ECs were also analyzed (HUVEC). (**A**) Cell viability assay performed at the indicated time points on infected HUVEC plated in 96-well plates and grown in complete medium. The optical density values were expressed as relative cell viability. (**B**) ECs were infected, plated in 24-well plates and grown in complete medium. Cell number was evaluated by using a hemocytometer at the indicated time points. (**C**) Migration assay on infected HUVEC. Cells were grown in growth factor-depleted culture medium and plated in a Boyden chamber. Chemotaxis was stimulated with 10 ng/ml VEGF (Sigma-Aldrich). Migratory cells were stained and counted under a light microscope. (**D** and **E**) Wound healing assays of HUVEC infected as indicated. Cell monolayers were wounded with a sterile pipette tip, washed, and grown in complete medium. Cells were observed under a light microscope and photographed at 0 and 8 h. Representative experiments are shown (original magnification, x100). (**F**) HUVEC infected as indicated were grown in complete medium on Matrigel and the formation of capillary-like structures was observed 20 h after seeding. A representative experiment is shown (original magnification, ×100). (**G**) Quantification of tube length was performed based on the results shown in panel F. Results were expressed as means ± SD of four different fields randomly chosen from each group. All data represent the means ± SD of three-five independent experiments.

Previously, we demonstrated that proliferation, migration, and differentiation of human primary ECs were strongly decreased when the function of CD93 was neutralized [5]. Therefore, to assess whether CD93/β-DG convergence had functional consequences in ECs during angiogenesis, we first analyzed changes in cell number and viability in DG-silenced HUVEC at different time points of cell growth. ECs infected with lentiviruses expressing either DG shRNA showed a decrease in cell viability, as well as in cell number when compared to cells not infected or infected with an unrelated shRNA (Figure 3A and 3B). Importantly, the same extent of reduction in cell number and viability was observed also in CD93-silenced cells (Figure 3A and 3B). Moreover, analysis of cell migration showed that ECs silenced for DG exhibited a significant decrease in VEGF-stimulated migration compared to control cells (Figure 3C), similar to that previously observed in CD93-silenced ECs [5]. Since in a wound healing assay the open gap is sealed through a combination of proliferation and migration [21], we asked whether CD93− or DG-silenced cells were able to heal a wound. As expected, HUVEC expressing either CD93 or DG shRNAs were unable to heal the wound in 8 hours of cell growth, in contrast to cells infected with an unrelated shRNA that filled the open gap in the same period of time (Figure 3D and 3E). Interestingly, proliferation and migration of CD93/DG double-silenced cells decreased in comparison to control cells and the extent of reduction was equal or higher to that observed for individual-silenced cells (Figure S4), suggesting that CD93 and β-DG exert unidirectional effects on downstream effector(s). Finally, we performed a tube formation assay on Matrigel, a substrate that allows attachment and differentiation of ECs. HUVEC infected with an unrelated shRNA formed a complete network of tubular-like structures, whereas only a small number of tubes were formed by DG-silenced ECs (Figure 3F and 3G). The same impaired tubulogenesis was previously described for HUVEC grown on Matrigel in the presence of an anti-CD93 neutralizing antibody [5].

### CD93 and DG crosstalk

Whilst DG is known to bind different ECM molecules [11], no ECM ligands have been identified for CD93 so far. To measure the direct binding of CD93 with ECM proteins, we produced soluble CD93 extracellular domain recombinant proteins and applied them to ELISA plates coated with different purified ECM proteins. No interactions were detected between CD93 and fibronectin, vitronectin, laminin, type I, or type IV collagen (Figure S5).

**Figure 4:**
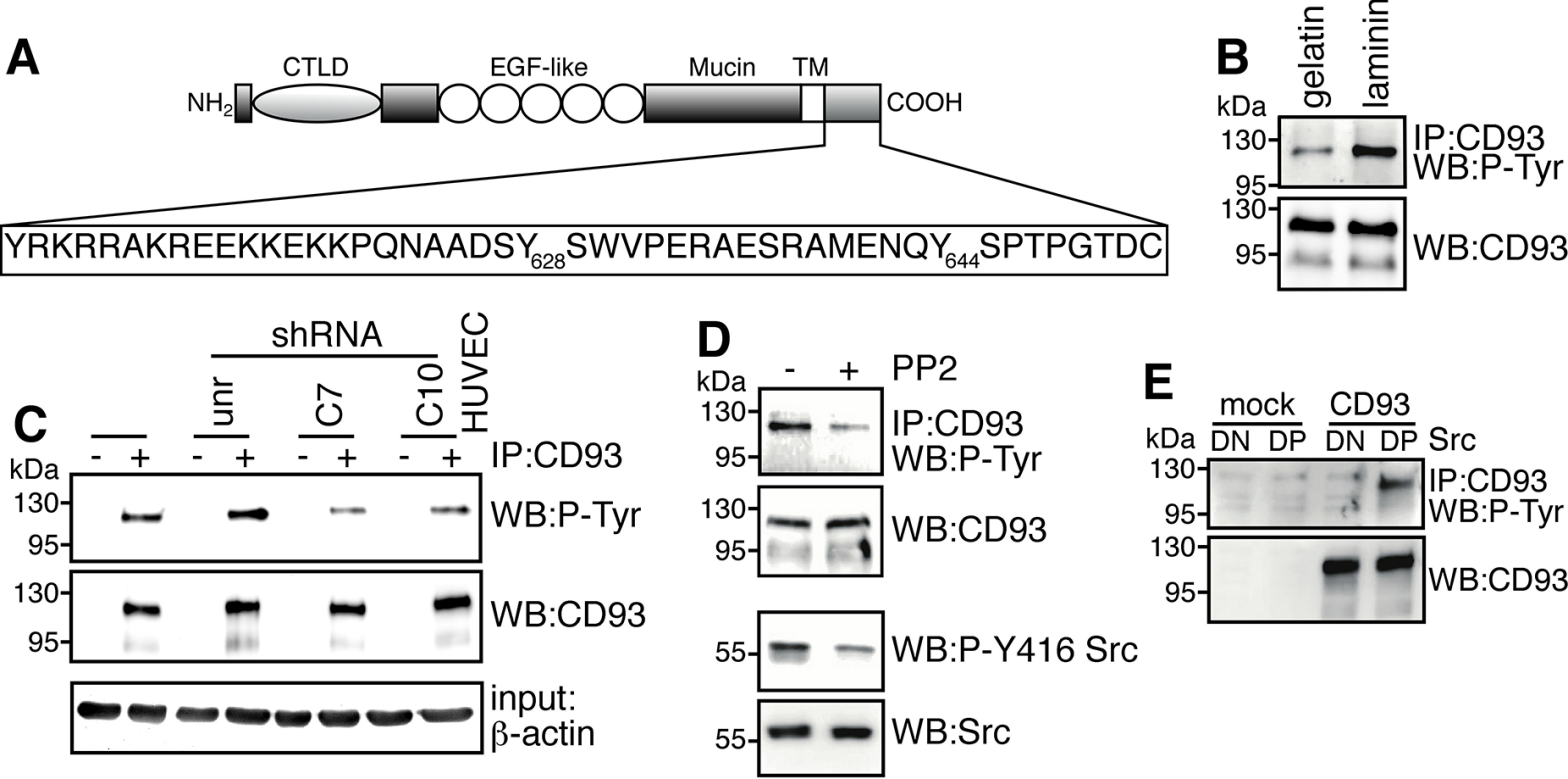
Binding of DG to laminin induces CD93 phosphorylation via Src (**A**) The schematic diagram illustrates the domains of CD93 and the 47-amino acid sequence of its cytoplasmic tail containing tyrosine 628 and 644. CTLD, C-type lectin-like domain; EGF-like, Epidermal Growth Factor repeats; Mucin. Mucin-like domain; TM, transmembrane domain. (**B**) Cell extracts from ECs spreading and growing on gelatin or laminin were immunoprecipitated with anti-CD93 antibodies. Immunoprecipitates were analyzed by Western blotting with anti-phosphotyrosine and anti-CD93 antibodies to confirm equal loading. (**C**) HUVEC were infected with a lentiviral vector expressing unrelated (unr) or DG (clones C7 or C10) shRNAs. Not infected ECs were also analyzed (HUVEC). Cell extracts from cells spreading and growing on laminin were immunoprecipitated or not (+, −) with anti-CD93 antibodies and analyzed by Western blotting with anti-phosphotyrosine antibodies. To confirm equal loading, whole cell lysates were analyzed by Western blotting with anti-CD93 and anti-β-actin antibodies. (**D**) HUVEC were allowed to adhere and grow on laminin in the presence (+) or not (−) of PP2 (10 μm). Cell lysates were immunoprecipitated with anti-CD93 antibodies and analyzed by immunoblotting with anti-phosphotyrosine and anti-CD93 antibodies to confirm equal loading. In the same cell lysates, phosphorylation on tyrosine 416 of Src, a protein modification that is closely correlated with kinase activity, was analyzed by Western blotting with anti-phospho-Y416 Src and anti-Src antibodies to confirm equal loading. All experiments were performed three-four times. (**E**) Human Lenti-X 293T cells, which do not express wild type CD93, were transiently cotransfected with a construct expressing human CD93 and the constitutively active (DP) or kinase dead (DN) Src kinase. Transfection with an empty vector (mock) is indicated. Cell lysates were immunoprecipitated with anti-CD93 antibodies and analyzed by Western blotting with anti-phosphotyrosine and anti-CD93 antibodies to confirm equal loading.

DG engagement by laminin results in the phosphorylation of the cytoplasmic domain of β-DG [22]. Since β-DG interacts with several proteins to transduce extracellular signals into cells and several cell adhesion processes are activated by tyrosine phosphorylation, we asked whether following cell adhesion the CD93/β-DG interaction could result in CD93 phosphorylation. To address this issue, we used computational analysis and identified two putative phosphorylation sites at the level of tyrosine 628 and 644 of the human sequence (Figure 4A). Interestingly, these tyrosine residues were found to be phosphorylated in a phosphoproteomic analysis of lung tumors [23]. Next, ECs were plated and allowed to spread onto laminin or gelatin. Cell extracts were immunoprecipitated with anti-CD93 antibodies and immunoblotting analysis using anti-phosphotyrosine antibodies revealed CD93 phosphorylation in cells spreading and growing on both substrates, even though CD93 phosphorylation levels were higher when cells were plated on laminin (Figure 4B). No CD93 phosphorylation was detected in cells plated in the absence of ECM components (data not shown). To investigate whether CD93 phosphorylation was due to DG engagement, ECs were silenced for DG and allowed to adhere and grow on laminin. DG knockdown caused a significant decrease of CD93 phosphorylation compared to control ECs (Figure 4C), suggesting that CD93 is activated in a signaling pathway triggered by DG adhesion to the substrate. Since it has been observed that following cell adhesion β-DG undergoes tyrosine phosphorylation creating a SH2 domain interaction site for the recruitment of active Src, which in turn phosphorylates other intracellular substrates [24, 25], we next examined the effect of PP2 on CD93 phosphorylation in HUVEC plated on laminin. Immunoprecipitation analysis showed that the treatment with PP2 impaired CD93 phosphorylation (Figure 4D). Moreover, the constitutively active but not the kinase dead Src kinase was able to phosphorylate CD93 (Figure 4E), indicating that Src can phosphorylate CD93.

### CD93 phosphorylation promotes EC adhesion and migration

**Figure 5:**
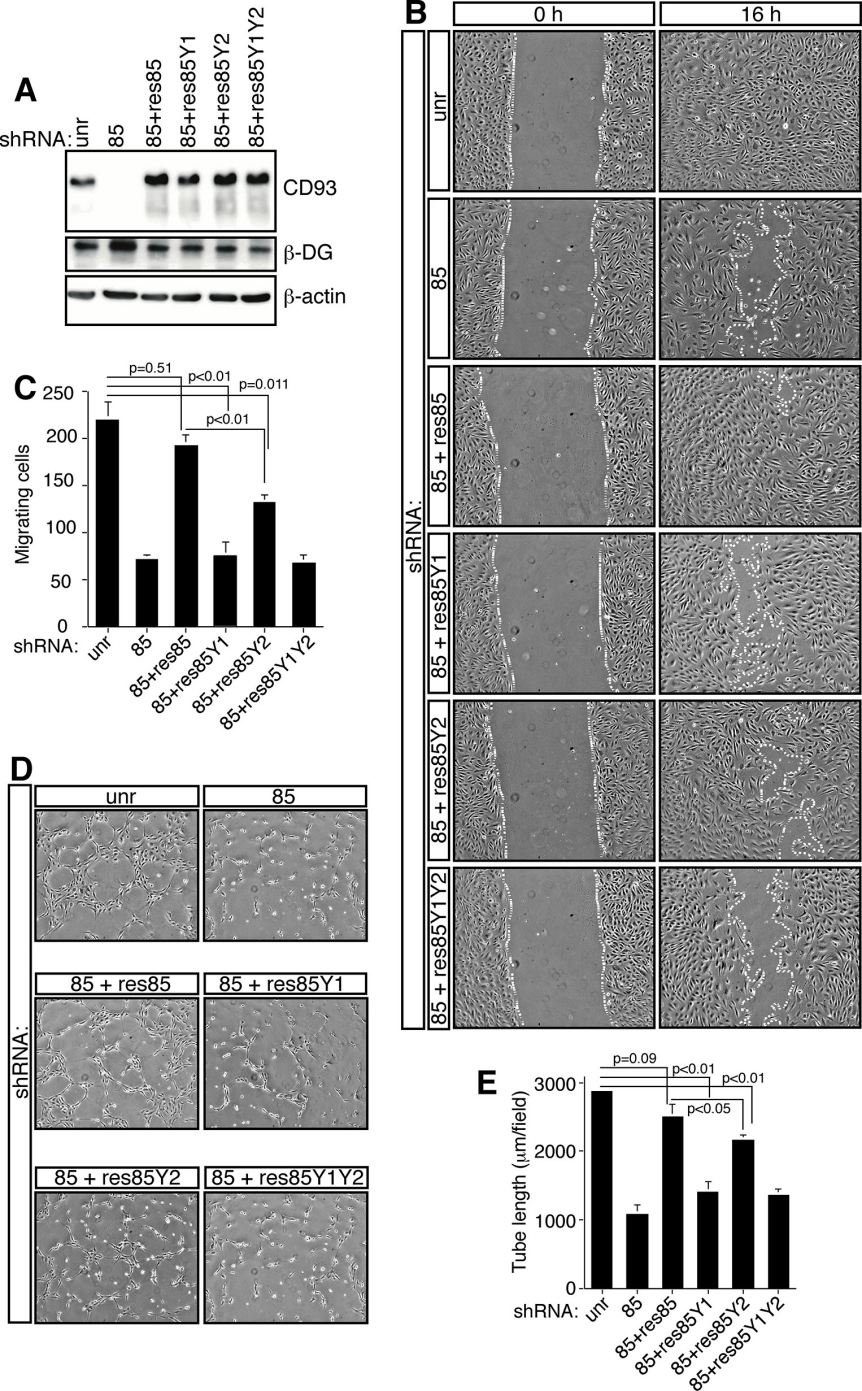
CD93 phosphorylation stimulates EC migration HUVEC were infected with lentiviral particles expressing CD93 shRNA clone 85 alone (85) or in combination (85+) with each CD93 mutant (res85, CD93 resistant to silencing; res85Y1, CD93 resistant to silencing mutated in tyrosine 628; res85Y2, CD93 resistant to silencing mutated in tyrosine 644; res85Y1Y2, CD93 resistant to silencing mutated in tyrosine 628 and 644). Control cells were infected with an unrelated shRNA (unr). (**A**) Cell extracts from infected ECs were analyzed by immunoblotting using anti-CD93, anti-β-DG, and anti-β-actin antibodies to confirm equal loading. (**B**) Wound healing assay of HUVEC infected as indicated. Cell monolayers were wounded with a sterile pipette tip, washed, and grown in complete medium. Cells were observed under a light microscope and photographed at 0 and 16 h. A representative experiment is shown (original magnification, ×100). (**C**) Migration assay on infected HUVEC. Cells were grown in growth factor-depleted culture medium and plated in a Boyden chamber. Chemotaxis was stimulated with 10 ng/ml VEGF. Migratory cells were stained and counted under a light microscope. (**D**) ECs infected as indicated were grown in complete medium on Matrigel and the formation of vascular capillary networks was observed 20 h after seeding. A representative experiment is shown (original magnification, ×100). (**E**) Quantification of tube length was performed based on the results shown in panel D. Results were expressed as means ± SD of four different fields randomly chosen from each group. All data represent the means ± SD of three independent experiments.

To elucidate the role of CD93 phosphorylation during angiogenesis, we produced CD93 expressing clones resistant to silencing and mutated in tyrosine residues of the cytoplasmic domain. We generated four mutants: the shRNA rescue (res85), expressing CD93 resistant to silencing by shRNA clone 85; the shRNA rescue mutated in tyrosine 628 (res85Y1); the shRNA rescue mutated in tyrosine 644 (res85Y2); and the shRNA rescue mutated in both phosphorylation sites (res85Y1Y2). Lentiviral particles obtained from CD93 mutant clones were used to transduce each mutant into HUVEC, silenced for endogenous CD93 by expression of the shRNA clone 85. As shown in Figure 5A, Western blotting analyses revealed that in CD93-silenced cells each infection restored CD93 at the same expression levels as observed in cells expressing an unrelated shRNA, whereas endogenous CD93 was completely knocked down. Moreover, the increased β-DG expression, as a consequence of CD93 depletion, was restored to control levels by expression of the CD93 mutants. In order to assess the role of CD93 phosphorylation in EC adhesion, we first analyzed the effects of mutant expression in cells spreading onto laminin. Even though CD93 phosphorylation mutant expressing ECs showed no gross morphological modifications during spreading (Figure S6A), the adhesion of these cells were significantly decreased in comparison to control cells or cells expressing the shRNA rescue mutant (Figure S6B). Next, we investigated the ability of HUVEC to heal a wound when we replaced endogenous with tyrosine-mutated CD93. HUVEC silenced for CD93 exhibited reduced migration, whereas the same cells expressing the shRNA rescue mutant restored their ability to fill the open gap. On the other hand, HUVEC expressing the phosphorylation mutants showed reduced migration similarly to CD93 silenced cells (Figure 5B). The altered migration phenotype was confirmed with a migration assay (Figure 5C). Further, HUVEC silenced for CD93 showed reduced ability to form a capillary-like network, whereas the same cells expressing the shRNA rescue mutant restored this ability. Importantly, ECs expressing the phosphorylation mutants showed reduced *in vitro* tube formation similar to CD93 silenced cells, even though CD93 mutant in tyrosine 644 showed a weaker reduction in tube formation (Figure 5D and 5E).

### CD93 signaling

**Figure 6:**
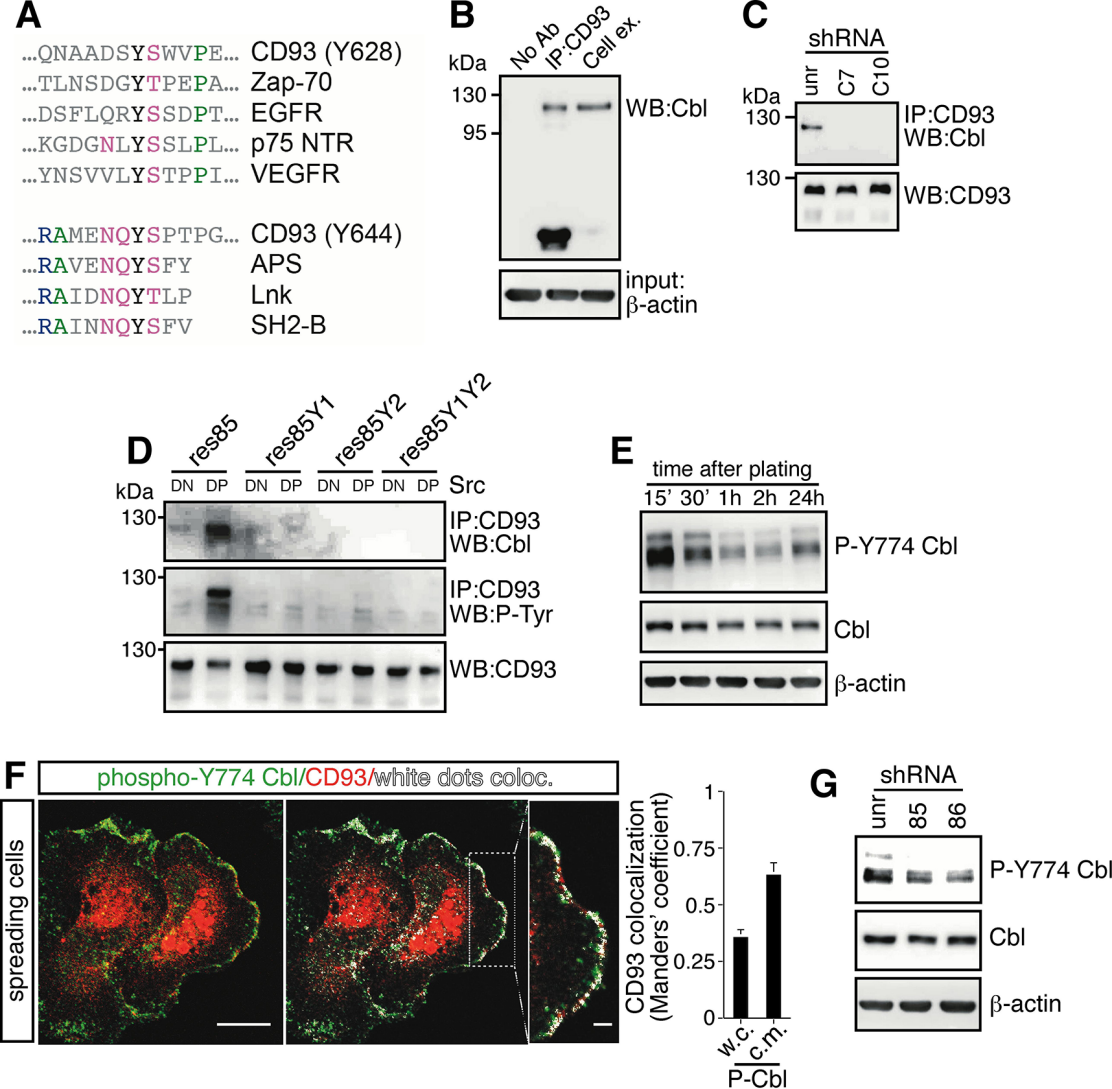
During EC spreading, Cbl is recruited to CD93 and phosphorylated on tyrosine 774 (**A**) Sequence alignment of Cbl recruitment sites previously characterized in different signaling proteins. Colored as opposed to gray residues are generally conserved. Green residues are hydrophobic, blue residues basic, and violet residues polar. Three dots indicate that the protein sequence continues. EGFR, epithelial growth factor receptor; NTR, neurotrophin receptor; VEGFR, vascular endothelial growth factor receptor; APS, adapter with pleckstrin homology and SH2 domains; Lnk, lymphocyte adaptor protein. APS, Lnk, and SH2-B belong to a family of adapter proteins that are implicated in signaling transduction [26]. (**B**) HUVEC were released from culture plates by EDTA treatment, plated on laminin-coated surface in complete medium, and allowed to spread. Cell lysates from spreading cells were immunoprecipitated with and without (No Ab) anti-CD93 antibodies. Immunoprecipitates were analyzed by Western blotting with anti-Cbl antibodies. Whole cell extracts were used to check the molecular size of the coimmunoprecipitated proteins (Cell ex.). Equal input was confirmed by Western blotting using anti-β-actin antibodies. (**C**) HUVEC were infected with a lentiviral vector expressing unrelated (unr) or DG (clones C7 or C10) shRNAs. Cell lysates were immunoprecipitated with anti-CD93 antibodies and analyzed by Western blotting with anti-Cbl and anti-CD93 antibodies to confirm equal loading. (**D**) Human Lenti-X 293T cells do not express wild type CD93 [5]. Cells were transiently cotransfected with constructs expressing CD93 mutants (res85, res85Y1, res85Y2, and res85Y1Y2) and the constitutively active (dominant positive, DP) or kinase dead (dominant negative, DN) Src kinase. Cell extracts were immunoprecipitated with anti-CD93 antibodies and analyzed by Western blotting with anti-Cbl, anti-phosphotyrosine, and anti-CD93 antibodies to confirm equal loading. In the presence of active Src, only wild type CD93 (res85) displays a phosphorylation signal and interacts with Cbl. (**E**) ECs were detached from culture plates and replated on laminin-coated surfaces. Cell extracts obtained at different degrees of cell spreading were analyzed by Western blotting using anti-phospho-Cbl(Y774) antibodies. 15′ and 2 h indicate early and late spreading cells respectively. 24 h after replating, cells reached confluency. (**F**) ECs were fixed during the spreading phases and analyzed by immunofluorescence using anti-CD93 and anti-phospho-Cbl(Y774) antibodies. Overlay of stained cells and white dot colocalization images are shown. Plot shows quantification (using Manders' coefficient) of CD93 colocalization with phospho-Cbl(Y774) at the cell margin (c.m.) and in whole cells (w.c.) (mean ± SD; cells = 20; *n* = 3). Scale bar, 14 μm. In the inset white dots show CD93 and phospho-Cbl(Y774) colocalization at the cell margin. Scale bar of the inset is 3 μm. (**G**) HUVEC were infected with lentiviral vectors expressing unrelated (unr) or CD93 (clones 85 or 86) shRNAs. Lysates obtained from spreading cells were analyzed by Western blotting using anti-phospho-Cbl(Y774) antibodies. In E and G to confirm equal protein loading, whole cell lysates were analyzed by Western blotting with anti-Cbl and anti-β-actin antibodies. Every experiment was repeated at least three times.

By computational analysis we observed that tyrosine 628 and 644 in the cytoplasmic domain of CD93 were contained in consensus sequences for the binding of the adapter protein Cbl (Figure 6A), which is recruited to target molecules through binding to tyrosine-phosphorylated sequences [26]. To verify the interaction between CD93 and Cbl, we performed coimmunoprecipitation experiments from lysates of spreading ECs. Antibodies against CD93 were able to coimmunoprecipitate Cbl from wild type cells (Figure 6B), whereas we did not observe this binding in β-DG-depleted cells (Figure 6C). Interestingly, coimmunoprecipitation analysis of CD93 mutants phosphorylated by Src kinase revealed that Cbl bound CD93 phosphorylated on tyrosine 628 and 644 (Figure 6D). Since tyrosine phosphorylation of Cbl has been shown to increase cell adhesion and spreading [27–29], we asked whether during EC adhesion Cbl was activated by phosphorylation. Western blotting analysis of EC lysates showed that during the spreading phase Cbl was strongly phosphorylated on tyrosine 774 (Figure 6E), while tyrosine 700 and 731 of Cbl were not (data not shown). Moreover, double immunofluorescence staining and colocalization analyses of spreading ECs showed that CD93 significantly colocalized with tyrosine 774-phosphorylated Cbl at the cell margin (Figure 6F). To assess whether Cbl phosphorylation on tyrosine 774 was due to the presence of CD93, we analyzed by Western blotting CD93-silenced cells, which showed reduced Cbl phosphorylation on tyrosine 774 as compared to control cells (Figure 6G). Taken together, these results indicate that following cell adhesion through DG and CD93 phosphorylation on tyrosine residues 628 and 644, CD93 binds to Cbl inducing phosphorylation on tyrosine 774 of Cbl.

## DISCUSSION

In this report, we describe the molecular interplay between CD93 and DG membrane proteins and highlight its importance in the improvement of EC adhesion and migration. Functional communication between distinct surface proteins has been formerly demonstrated and through their physical association and modulation of downstream effectors these molecules regulate cell physiology [17–19, 30]. We showed that CD93 and β-DG physically associated and that, following the imbalance caused by the absence of CD93, ECs overexpressed β-DG (and vice-versa) in an attempt to attain a normal physiological function, supporting a mechanism of close collaboration between CD93 and β-DG. Interestingly, the increased β-DG expression, as a consequence of CD93 silencing, was restored to the levels of control cells by CD93 mutant expression, suggesting that physical CD93/β-DG interaction is relevant for their balanced expression.

Our previous report has indicated that CD93 exerts its angiogenic function when ECs shuttle from a quiescent to a proliferating/migrating state [5]. Consistent with this observation, CD93 and also DG expression were found to be high in ECs of blood vessels inside cancerous in comparison to normal tissues [14, 31, 32], strengthening the complementary function of these proteins during formation of tumor vessels. Our study supports this concept of cooperation between CD93 and β-DG in the regulation of EC function during formation of new vessels. Indeed, by RNA interference technology, we observed that in ECs, CD93 or DG knockdown impaired to the same extent cellular physiologic and phenotypic characteristics, relevant to the angiogenic process.

The N-terminus of CD93 contains a C-type lectin-like domain (CTLD) [4]. Despite the fact that a previous study has reported the identification of fibronectin, collagen I, and IV as specific ligands for the CTLD of endosialin and demonstrated its role as an adhesion molecule in the regulation of cell migration during angiogenesis [33], we did not observe any interaction between CD93 and common ECM proteins. Although we cannot exclude that CD93 might bind different ECM proteins, we suggest that CD93, through DG adhesion to the substrate, activates a signaling pathway involved in EC adhesion and migration. Consistent with this requirement, we observed that following DG adhesion to laminin CD93 was phosphorylated by Src. Importantly, Src is the same kinase responsible for β-DG phosphorylation in response to cell adhesion, generating a DG recruitment site for active Src itself [24]. Moreover, analyzing CD93 mutants in phosphorylation sites we observed that CD93 phosphotyrosine 628 was predominantly instrumental for the stimulation of EC growth, adhesion and migration. This CD93 phosphotyrosine is located within a putative consensus motif for Cbl recognition. Cbl has been implicated in cell adhesion and organization of the actin cytoskeleton [29, 34], and its phosphorylation on tyrosine 774 provides a docking site for downstream signaling components, such as CrkL, which in association to Cbl, has been shown to enhance cell migration [28, 34, 35]. In agreement with these observations, we showed that in ECs Cbl directly bound to phosphorylated CD93 and was phosphorylated only on tyrosine 774. In addition, this Cbl phosphotyrosine colocalized with CD93 and was reduced in CD93-silenced cells, consistent with a role for CD93-dependent Cbl phosphorylation on tyrosine 774 in the enhancement of adhesion and migration. However, in the regulation of cytoskeletal phenomena Cbl can exert opposite effects following its interaction with the same protein by acting both as an adaptor and an E3 protein ligase [29, 34]. Interestingly, we observed that during cell spreading CD93 was ubiquitinated, but it was in a Cbl-independent manner (Figure S7), indicating that in this signaling pathway Cbl is mainly involved in the initial activation of a protein complex rather than in its subsequent inactivation.

Our study supports a model positing that CD93 and DG cooperate in the promotion of EC adhesion and migration during angiogenesis. Although speculative, we provide a molecular mechanism of how CD93 and DG crosstalk. When ECs interact with laminin, DG is phosphorylated creating a binding site for the recruitment of active Src. Then Src phosphorylates CD93 leading to the recruitment of Cbl. Finally, Cbl is phosphorylated on tyrosine 774, activating a signaling pathway involved in EC adhesion and migration. Consistent with this model, in platelet cells Cbl phosphorylation on tyrosine 774 is involved in adhesion and spreading and is inhibited in the presence of PP2 [36]. Moreover, DG has been involved in the formation of activating protein complexes responsible for podosome formation and spreading of myoblasts [25, 30]. In conclusion, since angiogenesis is activated in several pathological conditions and a combination of antiendothelial drugs is an attractive therapeutic modality [37], our study, identifying a new signaling pathway based on the cooperation between CD93 and β-DG, may lead to the discovery of potential targets to be used in antiangiogenic therapy.

## MATERIALS AND METHODS

### DNA plasmids and constructs

The full-length cDNA of human *DG* (GenBank^™^ accession number NM_004393) was amplified using the BcaBEST^™^ RNA PCR kit (Takara Bio Inc., Otsu, Japan) from reverse transcription of total RNA extracted from HUVEC (oligonucleotides M479, 5′-GAGAA AGCTTGCCACCATGAGGATGTCTGTGG-3′ and M203, 5′-GAGAGACTCGAGTTAAGGTGGGACATAGG-3′) and cloned in pcDNA3 cloning vector (Thermo Fisher Scientific, Waltham, MA, USA). The full-length cDNA of human *CD93* was cloned as previously described [5]. For FRET analysis *CD93* and *DG* full-length cDNA were subcloned into pEYFP-N1 and pECFP-N1 (Clontech Lab, Mountain View, CA, USA) vectors respectively, positioning the fluorescence tags at C terminal. The c-Src expression vectors were obtained as previously described [38]. The CD93 mutants were obtained by using the QuickChange II XL Site-Directed Mutagenesis kit (Agilent Technologies, Santa Clara, CA, USA) according to manufacturer's instructions. Briefly, full-length cDNA of human CD93 was subcloned into pCCL retroviral vector [39]. The shRNA mutant (rescue) resistant to the clone 85 of CD93 shRNA was obtained by introducing a silent mutation in the shRNA recognition sequence by the exchange (TCT to AGC) of the serine codon 126. The CD93 tyrosine (628 and 644) to phenylalanine substitutions were obtained by changing the codon sequences from TAC to TTC. All constructs were confirmed by sequencing.

### 2-DE and mass spectrometry

Cells were detached from culture plates by scraping in PBS containing EDTA, washed twice with ice-cold PBS, resuspended in a buffer containing 65 mM DTE, 65 mM CHAPS, 9 M urea, 35 mM Tris-base, and disrupted by sonication in an ice bath. 60 μg of proteins/sample were submitted to 2-DE as previously described [40]. Digitalized images were obtained by ImageScanner III and then qualitatively and quantitatively analyzed by the ImageMaster software (GE Healthcare BioSciences, Piscataway, NJ, USA). The increasing/decreasing index (fold change) was calculated as the ratio of spot relative volume between the different gel maps. Protein spot identification was obtained by MALDI-ToF/MS as previously described [41]. Mascot software v.2.2 (Matrix Science, Boston, MA, USA) was used to identify spots from the NCBI non-redundant database. Candidates with a score > 81 (corresponding to *p* ≥ 0.05 for a significant identification) were further evaluated by comparison with experimental coordinates from 2-DE.

### Cell culture and transfection

HUVEC were isolated from umbilical cords collected from consenting healthy patients according to institutional guidelines. For each experiment at least three independent extractions of HUVEC were used. Cells were cultured on gelatin-coated Petri dishes as previously described [42]. Transient transfection experiments were performed by electroporation. Subconfluent cells were detached from the culture dish by trypsin treatment, washed, and resuspended in Ham's F12 culture medium (Thermo Fisher Scientific) containing 2.5% fetal bovine serum at a concentration of 4 × 10^6^ cells/cuvette with 20 μg of plasmid DNA. Cells were electroporated with one 0.15-ms pulse of 290 V (Gene Pulser II, Bio-Rad Laboratories, Hercules, CA, USA). After an additional incubation for 5 min at 37°C, cells were plated on appropriate culture dishes. Human Lenti-X 293T (Clontech Lab) and mouse BALB/c cell lines were cultured using standard conditions. In these cell lines transient transfection experiments were performed using Lipofectamine 2000 (Thermo Fisher Scientific) according to manufacturer's instructions.

### Immunofluorescence microscopy

Cells were seeded on glass coverslips, fixed in 3% paraformaldehyde, and then treated as previously described [43]. The primary antibodies used were: mouse monoclonal anti-CD93 (mAb 4E1, 0.6 μg/μL [5]) 1:25, rabbit anti-β-DG (Santa Cruz Biotechnology, Santa Cruz, CA, USA), and rabbit anti-phospho-Cbl (Y774) (Millipore, Billerica, MA, USA). Fluorescent images were captured using a Leica TCS SP2 laser-scanning confocal microscope. The quantitative colocalization analyses of CD93 and β-DG or phospho-Cbl signals were performed on optical sections captured at cell/substrate adhesion sites using ImageJ and the JACoP plug-in to determine Manders' coefficient M_1_ [44], which represents the percentage of CD93 pixels that overlap β-DG or phospho-Cbl pixels. To show colocalization events by white dots, images were generated using ImageJ and the Colocalization plug-in.

### Protein-protein interaction analyses

FRET acceptor photobleaching (apFRET) was carried out on fixed cells as previously described [45, 46]. Briefly, HUVEC transiently transfected and co-expressing β-DG-CFP and CD93-YFP were analyzed by using a 40 mW argon laser line of 458 nm to excite β-DG-CFP (PMT window 465–505) and a line of 514 to excite CD93-YFP (PMT window 525–590). Cells transfected with a single construct were used to calibrate laser intensity to prevent bleed-through. Acquisition parameters include image size of 512 × 512 pixels, 400Hz line scanning-rate, zoom 4x. In the photobleaching procedure, cells were bleached using a 514 nm laser beam at 100% intensity until the acceptor was photobleached down to about 10% of its initial value. The bleach time ranged from 2 to 5 s. For each experiment, data were collected from 25–30 different cells in different fields. For FRET analysis, we selected about half-cell as region of interest (ROI) and, after photobleaching, additional ROIs were chosen inside the photobleached area, which included both more discrete cell regions and an outside of the cell to use as background. FRET measurement was performed using the apFRET software (Leica Microsystems, Wetzlar, Germany), according to the manufacturer's instructions.

The *in situ* interaction between CD93 and β-DG was detected by using the Duolink II detection kit (Olink Bioscience, Uppsala, Sweden) following the manufacturer's instructions. Briefly, exponentially growing HUVEC were treated as for immunofluorescence microscopy by using mouse anti-CD93 and rabbit anti-β-DG antibodies. Duolink secondary antibodies, provided as conjugates to oligonucleotides that are ligated together in a closed circle if the primary antibodies are in close proximity, were added. Polymerase amplification of any existing closed circles was performed and detection was achieved with complementary, fluorescently labeled oligonucleotides. Background was assayed by removing one of the two primary antibodies from the reaction. Fluorescent images were acquired under a confocal microscope at the focal plane of cell/ECM contact.

### RNA interference-mediated knockdown of CD93 and DG

Silencing experiments were performed using retroviral vectors pLKO.1 from the TRC lentiviral shRNA library (Open Biosystems, Huntsville, AL, USA) expressing specific shRNAs for human CD93 [5], and DG (oligonucleotide TCRN0000056188, referred to as C7; and oligonucleotide TCRN0000056191, referred to as C10). Recombinant lentiviruses were produced and used for infection experiments as previously described [47].

### Cell viability, adhesion, and motility assays

HUVEC viability and cell adhesion were evaluated as previously described [5]. For the wound healing assay, 1.5 × 10^5^ HUVEC were seeded in a 24-well plate coated with gelatin and cultured for 24 hours. When cells reached the confluence, a straight scratch was created in the monolayer using a sterile pipette tip. Cultures were washed with PBS and grown in complete medium. Bright-field images were captured using an inverted microscope (Axiovert 200, Zeiss, Oberkochen, Germany) equipped with a Nikon DXM1200 digital camera. For each condition, images were acquired at three different positions along the scratch and a representative field was depicted. Chemotaxis analysis with Boyden transwell chambers and formation of capillary-like tube structures in Matrigel were performed as previously described [48].

### Immunoprecipitation and immunoblotting analyses

Immunoprecipitation and immunoblotting experiments were performed as previously described [49]. For co-immunoprecipitation experiments cells were lysed in Co-ip buffer (1% NP-40, 150 mM NaCl, 10 mM Tris-HCl pH 7.5, 1 mM EDTA, protease and phosphatase inhibitors, Sigma-Aldrich, St Louis, MO, USA). The following antibodies were used for immunoblotting: rabbit anti-CD93 (H190), mouse anti-phosphotyrosine, mouse anti-ubiquitin, and rabbit anti-β-DG (Santa Cruz Biotechnology); mouse anti-Cbl (Millipore); rabbit anti-phospho-Cbl(Y731), rabbit anti-phospho-Y416 Src, and rabbit anti-Src 32G6 (Cell Signaling Tech., Danvers, MA, USA); rabbit anti-phospho-Cbl (Y700) (Abcam, Cambridge, UK); mouse anti-β-actin (Sigma-Aldrich). For immunoprecipitations, mouse anti-CD93 antibodies (mAb 4E1) were used coupled to Dynabeads Pan Mouse IgG (Thermo Fisher Scientific). The Src family tyrosine kinase inhibitor PP2 was purchased from Calbiochem (San Diego, CA, USA).

### Statistical analysis

All results are presented as means ± SD of at least three independent experiments. Statistical analysis was performed using Student's *t*-test and values of *p* ≤ 0.05 were considered statistically significant.

## SUPPLEMENTARY MATERIALS FIGURES



## Abbreviations

The abbreviations used are:

EC: endothelial cell
HUVEC: human umbilical vein endothelial cells
DG: dystroglycan
ECM: extracellular matrix
FRET: fluorescence resonance energy transfer
2-DE: 2-dimensional electrophoresis
