# Supplementary material for: CD93 and dystroglycan cooperation in human endothelial cell adhesion and migration
Source: Oncotarget. 2016 Feb 2;7(9):10090–103. doi: 10.18632/oncotarget.7136 (PMC4891106; doi:10.18632/oncotarget.7136)
Supplement: Supplementary file 1 [file oncotarget-07-10090-s001.pdf]

CD93 and dystroglycan cooperation in human endothelial cell adhesion and migration

Supplementary Materials

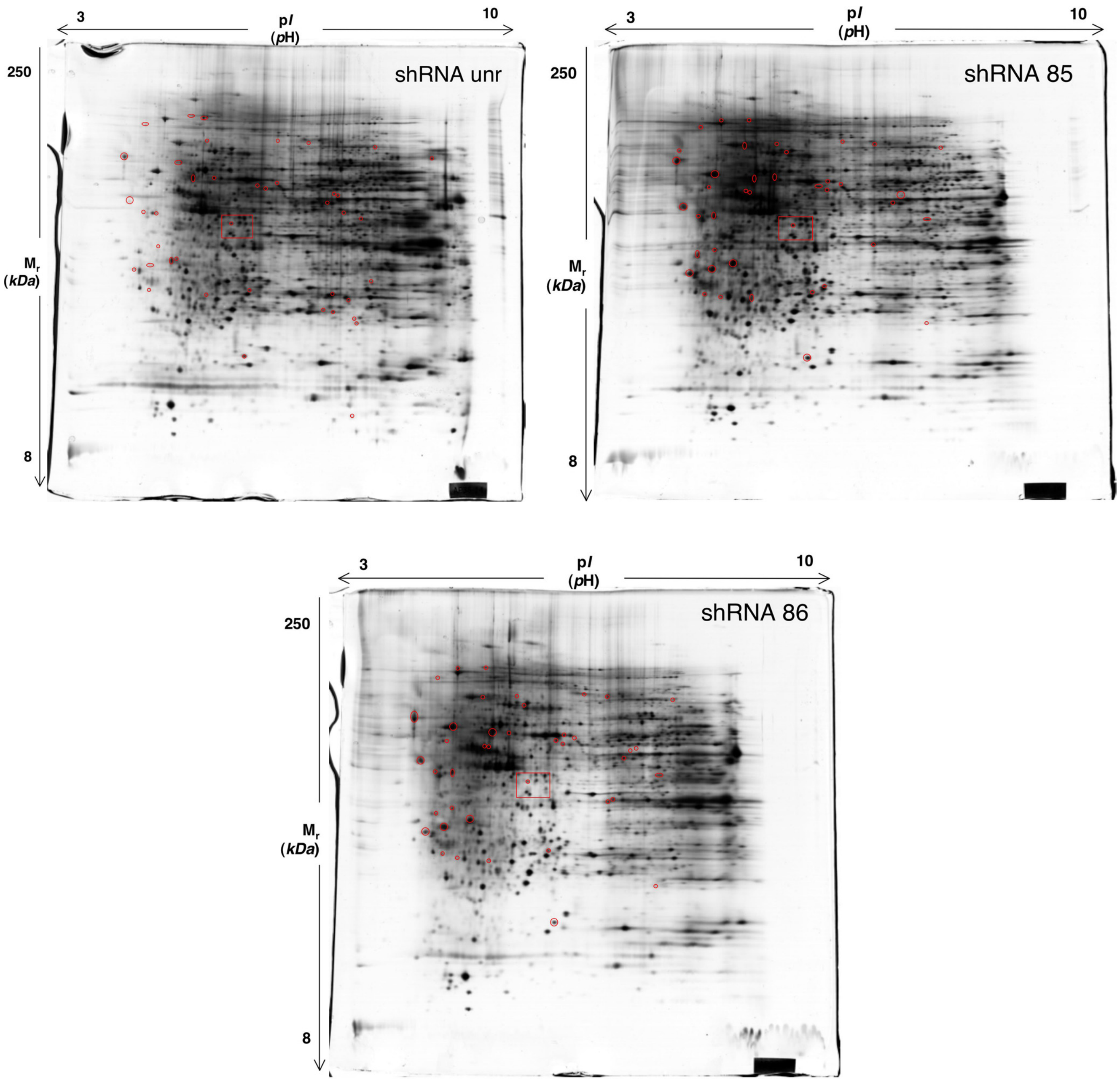

Supplementary Figure S1: In ECs CD93 silencing modifies protein expression. Silver stained 2-DE gels of total proteins extracted from HUVEC infected with lentiviruses expressing: (**A**) an unrelated shRNA; (**B**) CD93 shRNA clone 85; (**C**) CD93 shRNA clone 86. Representative images from a triplicate set are shown. Proteins differentially expressed are circled and the box indicates the expanded area shown in Figure 1.

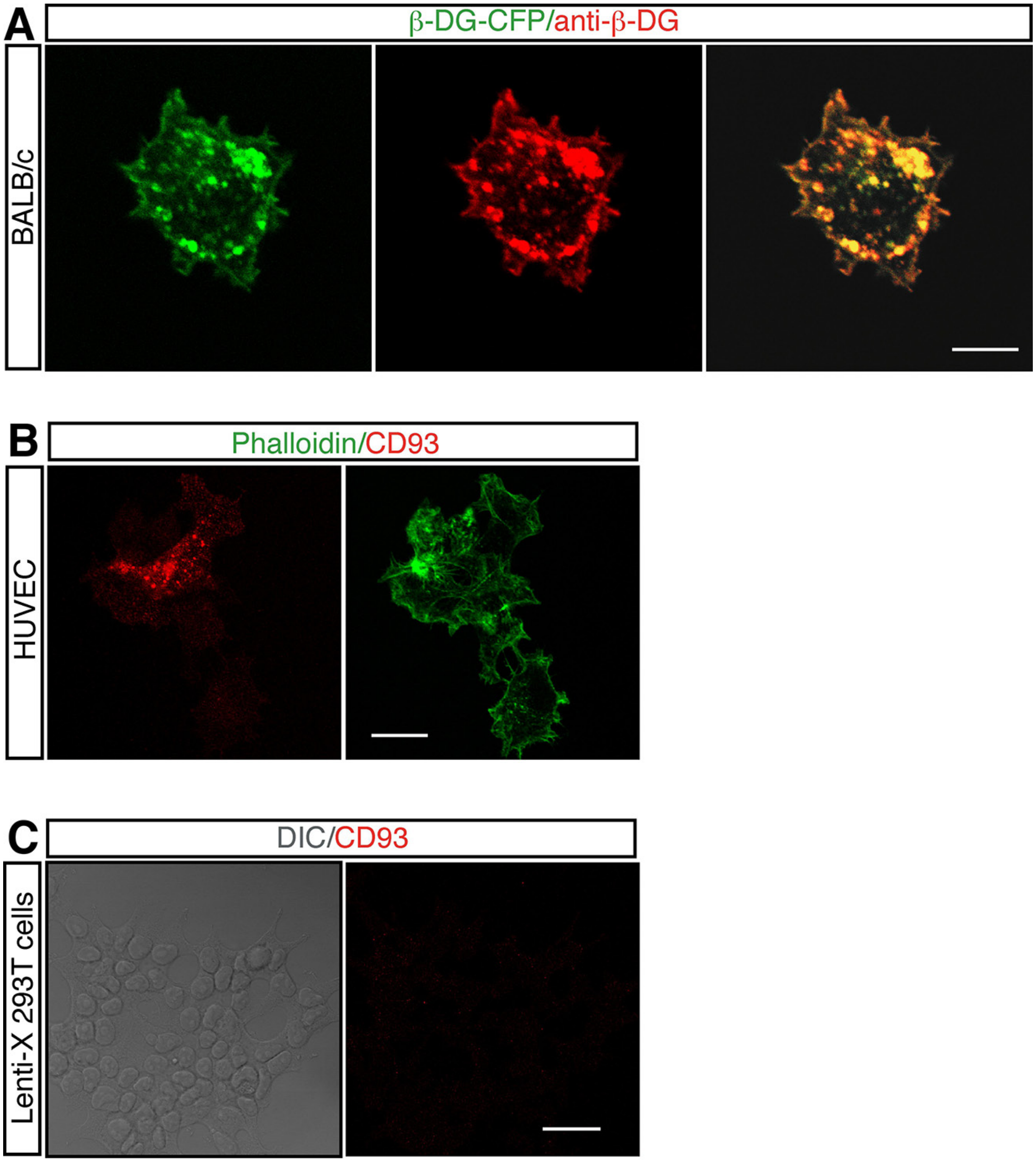

Supplementary Figure S2: Characterization of the anti-β-DG and anti-CD93 antibodies. (**A**) Immunofluorescence analysis on mouse BALB/c fibroblasts transfected with a construct expressing human β-DG-CFP under the control of a constitutive promoter. Transfected cells were plated on glass coverslips, fixed, permeabilized and analyzed using anti-β-DG antibodies that crossreact with β-DG of mouse and human origin. Antibody staining overlapped entirely over β-DG-CFP, indicating that the exogenously expressed protein is indeed β-DG and β-DG-CFP is correctly processed and transported to the plasma membrane. Scale bar, 7 µm. (**B**) HUVEC, infected with a lentiviral vector expressing a CD93 shRNA (clone 85), were mixed together with not infected cells and plated on glass coverslips to obtain cells expressing or not CD93 in the same microscope field. Cells were analyzed by immunofluorescence using anti-CD93 antibodies and Alexa Fluor-488 phalloidin (Thermo Fisher Scientific) for F-actin labeling. A representative image shows no staining in CD93-depleted cells compared to wild type cells. Scale bar, 18 µm. (**C**) Human Lenti-X 293T cells were analyzed by immunofluorescence using anti-CD93 antibodies. DIC image of stained cells is shown. Scale bar, 35 µm. Negative staining indicates that the anti-CD93 monoclonal antibody 4E1 does not crossreact with other proteins in human cells.

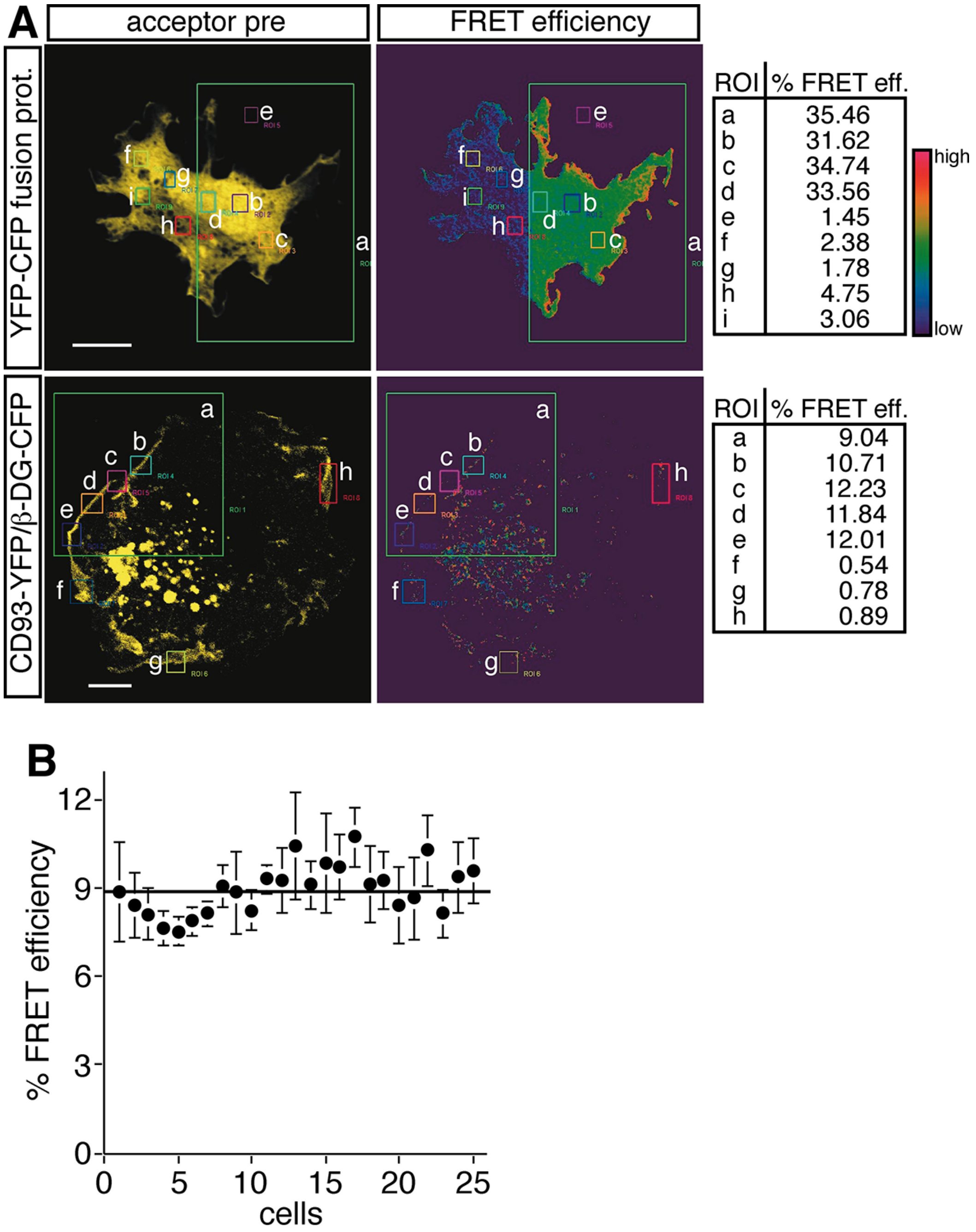

Supplementary Figure S3: FRET between CD93 and β-DG. (**A**) Representative confocal images of the YFP channel emission before photobleaching (acceptor pre) and FRET efficiency occurring when β-DG-CFP is coexpressed with CD93-YFP in HUVEC. Cotransfected cells were plated on laminin-coated coverslips and fixed during cell spreading. A selected cell region of CD93-YFP was chosen and photobleached using the 514 nm laser line. The expression of the β-DG-CFP alone showed that photo-conversion was not occurring in our experimental conditions (data not shown). Tables report the values of FRET efficiency calculated for additional ROIs within and outside the cell (in this case to use as background). The maximum level of apFRET obtained in our imaging conditions by measuring the FRET efficiency using the YFP-CFP fusion protein corresponded to 31.5 ± 3.9%. The colored scale represents the color range of FRET efficiency. Scale bars, 12 μm. (**B**) A representative plot showing β-DG-CFP fluorescence increased (% of FRET efficiency) upon CD93-YFP photobleaching measured in 25 different ECs. For each cell subjected to apFRET, the background was subtracted from the mean value of the photobleached additional ROIs. In this plot, the straight line indicates the average of FRET efficiency calculated for every cell (8.96 ± 0.84%). All data represent the means ± SD of three independent experiments, carried out on different days and with different cell preparations.

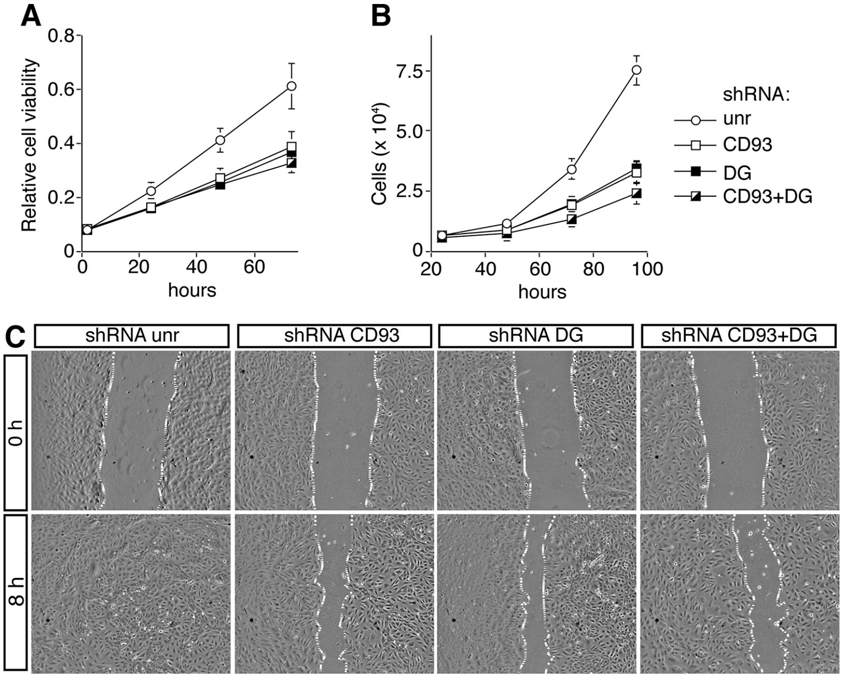

Supplementary Figure S4: CD93/DG double knockdown impairs EC proliferation and migration. HUVEC were infected with a lentiviral vector expressing unrelated (unr), or CD93 (clone 85), or DG (clone C7), or CD93+DG (clones 85 plus C7) shRNAs. (**A**) Cell viability assay performed at the indicated time points on infected HUVEC plated in 96-well plates and grown in complete medium. The optical density values were expressed as relative cell viability. (**B**) ECs were infected, plated in 24-well plates, and grown in complete medium. Cell number was evaluated by using a hemocytometer at the indicated time points. (**C**) Wound healing assay of HUVEC infected as indicated. Cell monolayers were wounded with a sterile pipette tip, washed, and grown in complete medium. Cells were observed under a light microscope and photographed at 0 and 8 h. A representative experiment is shown (original magnification, ×100). All data represent the means ± SD of three independent experiments each performed in triplicate.

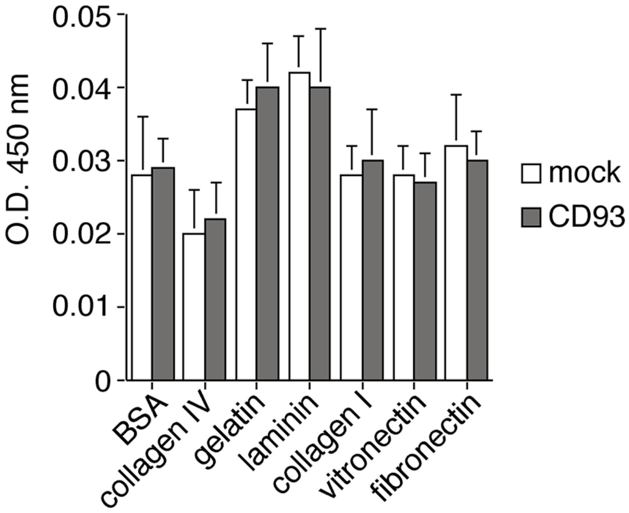

Supplementary Figure S5: The extracellular domains of CD93 do not bind ECM proteins. The chimeric construct containing the extracellular domains of CD93 fused to Myc tag was generated as previously described [5]. Human Lenti-X 293T were transiently transfected with empty vector (mock) or the construct expressing the soluble extracellular domains of CD93 (CD93). 72 h after transfection, conditioned media were collected and the concentration of the recombinant proteins was estimated by Western blotting and adjusted at about 0.8 μg/mL. The following adhesive substrates were dissolved in PBS and used for coating of 96-well plates (Nunc Maxisorp, Thermo Fisher Scientific): human vitronectin (1 μg/mL) from Thermo Fisher Scientific, rat collagen (from tail tendon, mainly type I collagen, 10 μg/mL) from Roche Diagnostics (Mannheim, Germany), and porcine gelatin (1%), bovine serum albumin (1%), mouse laminin (10 μg/mL), human fibronectin (10 μg/mL), and human type IV collagen (10 μg/mL) from Sigma-Aldrich. Plates were blocked with bovine serum albumin (3% in PBS) before the addition of supernatants. Plates were washed and HRP-conjugated mouse anti-Myc antibody (9E10, Santa Cruz Biotechnology) was added to detect bound CD93. o-phenylenediamine (Sigma-Aldrich) was used to asses color development. The optical density values were measured at 450 nm using a microtiter plate reader.

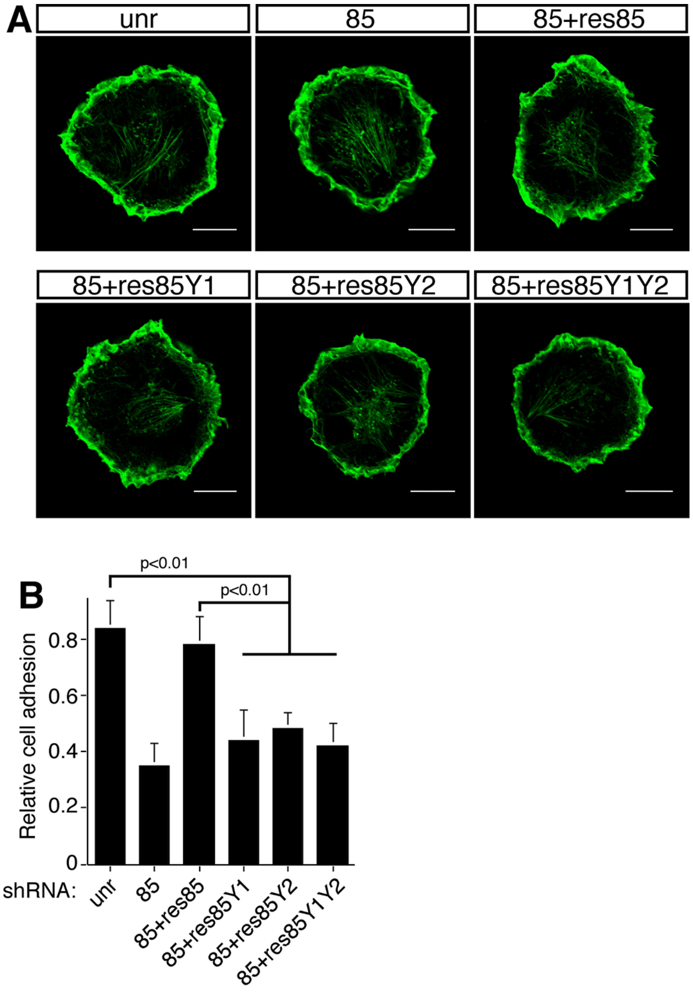

Supplementary Figure S6: Effects of CD93 phosphorylation mutant expression on EC spreading and adhesion. HUVEC were infected as indicated in Fig. 5. Infected cells were detached from culture plates by EDTA treatment, plated on laminin-coated surfaces in complete medium, and allowed to adhere. A: Cells were fixed at 15, 30, and 45 min after plating. Alexa Fluor-488 phalloidin was used for F-actin labeling. Representative fluorescent images captured with a confocal microscope at 30 min are shown. Scale bars, 13 µm. B: In cell adhesion assays, cells were fixed at 15 min after plating, stained with crystal violet solution and the optical density values were expressed as relative cell adhesion. Data represent the means ± SD of three independent experiments each performed in triplicate.

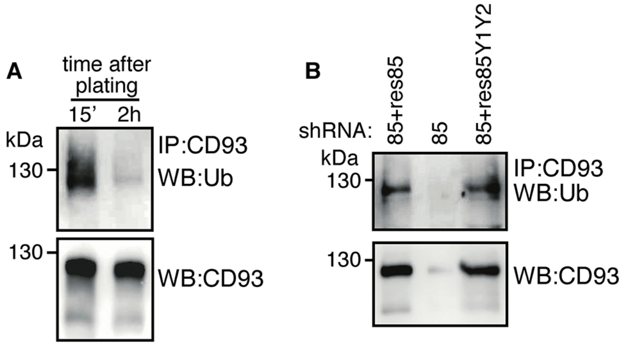

Supplementary Figure S7: In early spreading ECs CD93 ubiquitination is independent of Cbl. (**A**) ECs were released from culture plates and replated on laminin-coated surfaces. Cell extracts obtained at different degrees of cell spreading were immunoprecipitated with anti-CD93 antibodies and analyzed by Western blotting with anti-ubiquitin and anti-CD93 antibodies to confirm equal loading. 15′ and 2 h indicate early and late spreading cells respectively. (**B**) HUVEC were silenced for CD93 by using lentiviral particles expressing shRNA 85. Silenced cells were infected with lentiviruses expressing CD93 mutants (res85 and res85Y1Y2) and plated on laminin-coated surfaces. Cell extracts from early spreading cells were immunoprecipitated with anti-CD93 antibodies and analyzed by Western blotting with anti-ubiquitin and anti-CD93 antibodies to confirm equal loading.
